# Factors regulating capillary remodeling in a reversible model of inflammatory corneal angiogenesis

**DOI:** 10.1038/srep32137

**Published:** 2016-08-26

**Authors:** Anthony Mukwaya, Beatrice Peebo, Maria Xeroudaki, Zaheer Ali, Anton Lennikov, Lasse Jensen, Neil Lagali

**Affiliations:** 1 Department of Ophthalmology, Institute for Clinical and Experimental Medicine, Faculty of Health Sciences, Linköping University, 58183 Linköping, Sweden; 2 Department of Medical and Health Sciences, Division of Cardiovascular Medicine, Linköping University, 581 83 Linköping, Sweden

## Abstract

Newly formed microcapillary networks arising in adult organisms by angiogenic and inflammatory stimuli contribute to pathologies such as corneal and retinal blindness, tumor growth, and metastasis. Therapeutic inhibition of pathologic angiogenesis has focused on targeting the VEGF pathway, while comparatively little attention has been given to remodeling of the new microcapillaries into a stabilized, functional, and persistent vascular network. Here, we used a novel reversible model of inflammatory angiogenesis in the rat cornea to investigate endogenous factors rapidly invoked to remodel, normalize and regress microcapillaries as part of the natural response to regain corneal avascularity. Rapid reversal of an inflammatory angiogenic stimulus suppressed granulocytic activity, enhanced recruitment of remodelling macrophages, induced capillary intussusception, and enriched pathways and processes involving immune cells, chemokines, morphogenesis, axonal guidance, and cell motility, adhesion, and cytoskeletal functions. Whole transcriptome gene expression analysis revealed suppression of numerous inflammatory and angiogenic factors and enhancement of endogenous inhibitors. Many of the identified genes function independently of VEGF and represent potentially new targets for molecular control of the critical process of microvascular remodeling and regression in the cornea.

In the eye, pathologic angiogenesis is linked to blindness, which in the retina can result from age-related macular degeneration (AMD), retinopathy of prematurity (ROP)^1^ and diabetic retinopathy which is also associated with retinal vein occlusion (RVO)^2^. In the cornea, abnormal blood vessel growth following surgery, infection, or injury can lead to vision loss^3^ through scarring, inflammation, edema, and transplant rejection.

Vascular endothelial growth factor (VEGF) has been studied extensively, mainly as a potent vasodilator and an angiogenic guidance molecule for newly formed capillary sprouts and is necessary for their survival^4^. This role of VEGF has led to the development of anti-VEGF therapy designed to regress immature vessels^5^, with applications in both oncology and ophthalmology, with therapeutic success reported^6^,^7^,^8^,^9^. Despite the benefits of anti-VEGF therapy, it has been reported to have neurotoxic side effects, increase the risk of cerebral thromboembolic events (stroke)^10^, and to be only partially effective in regressing new vessels^3^,^11^. Reduction in efficacy may partly be attributed to an accelerated rate of remodeling and maturation processes that newly formed capillaries undergo, rendering them nonresponsive to anti-VEGF therapy (acquired VEGF-resistance)^12^,^13^. Additionally, not only VEGF but numerous pro-angiogenic factors can independently stimulate corneal angiogenesis^14^.

Alternative pathways promoting pathological angiogenesis and a better understanding of factors influencing capillary remodeling are therefore of therapeutic interest. At the microscopic level, remodeling has been shown to involve different cell types. In prior work, blood vessel regression in the rat cornea was characterized by macrophages closely associated with degrading capillary walls^15^. In the retina, disruption of endothelial-pericyte associations results in excessive retinal vessel regression and abnormal remodeling during hyperoxia treatment in mice^16^. Remodeling of extracellular matrix (ECM) has also been found to be spatiotemporally linked to vessel regression, as cleavage of endothelial cell (EC)-ECM integrin contacts critically influences EC survival^17^. In the zebrafish, it has been shown that blood vessel regression involves EC rearrangements, lumen collapse and intercellular contact resolution^18^.

Several genes have been associated with vessel remodeling and stabilization. PDGF and Ang1 are reported to be responsible for pericyte recruitment to stabilise newly formed vasculature^19^,^20^. In addition to EC tip selection, DLL4/Notch are reported to be associated with vessel pruning and regression^21^,^22^. Notch signalling is also important for vein and perivenous capillary plexus remodeling^23^. ANG/TIE signalling has also been documented to play a role in vessel pruning and regression^24^. The interaction between CXCL10 and CXCR3 (expressed by ECs) is key for disruption of integrin contacts, EC detachment, and apoptosis in dermal wound healing^25^. EC specific genes like FGD5 have been reported to induce HEY/p53 signalling leading to VEGF sequestration by increasing the VEGFR1/VEGFR2 ratio, resulting in EC apoptosis and vessel regression^26^. Additionally, the WNT signalling enhancer R-spondin3 (RSPO3) expressed by ECs has recently been identified to be crucial for maintaining a remodeling vasculature^27^.

In many clinical situations, however, angiogenesis occurs in a complex pro-inflammatory environment, such as in corneal transplant rejection^28^, in age-related macular degeneration^29^, and in the tumor microenvironment^30^. A host of inflammatory factors may therefore modulate microvasculature maturation and remodeling. A model of angiogenesis in the presence of significant inflammation would therefore mimic the complex physiologic situation and may reveal important inflammatory mediators of the angiogenic remodeling process.

In this study, we therefore sought to examine the process of capillary remodeling in an inflammatory context in the cornea. Besides mimicking clinical situations where inflammation plays an important role, the corneal model presents a unique opportunity to investigate angiogenesis in a normally avascular tissue possessing endogenous mechanisms for maintaining and regaining avascularity. We therefore employed a reversible model of corneal angiogenesis and longitudinally examined cells and microvessels *in vivo* to elucidate the dynamics of reversal of angiogenesis and restoration of a non-inflammatory and stable microenvironment at the tissue level. Whole-transcriptome profiling was then applied to identify known and potentially novel endogenous factors regulating the return to homeostasis – factors that could provide promising future targets to modulate inflammation, angiogenesis, and capillary remodeling. We hypothesized that the use of this reversible model would reveal key endogenous factors regulating inflammation and angiogenic remodeling independent of VEGF.

## Results

### Vascular density reduction is accelerated and basement membrane deposition is delayed by reversal of an angiogenic stimulus

**Figure 1:**
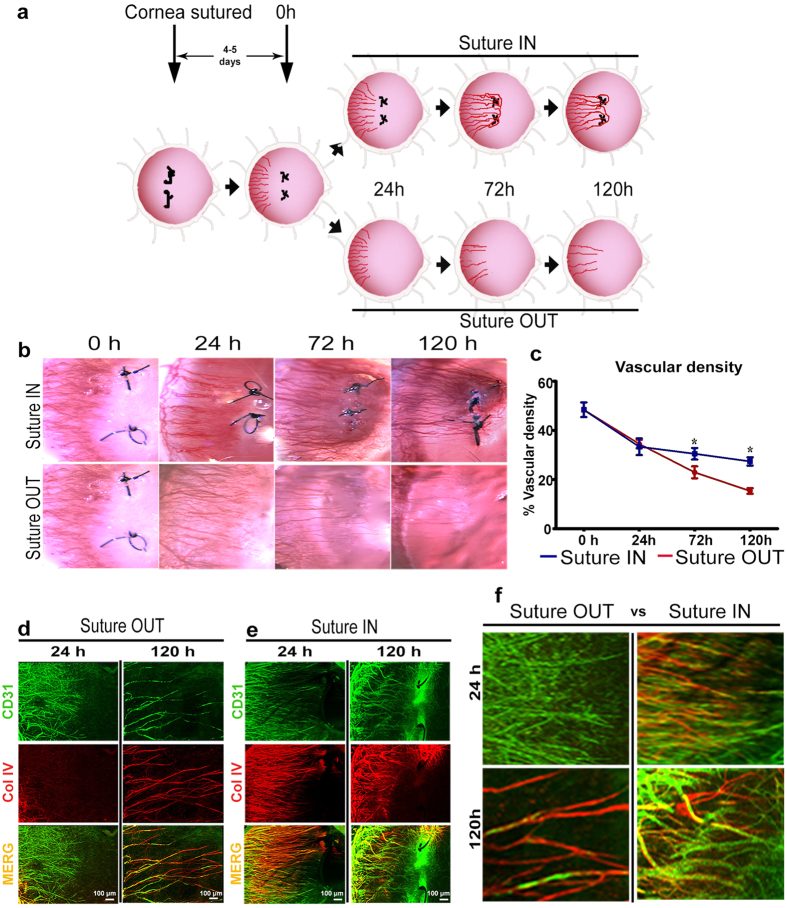
Vascular remodeling is accelerated by suture removal. (**a**) Reversible suture-induced inflammatory corneal neovascularisation model used for the study. (**b**) Serial slit lamp images of suture IN and suture OUT arms. (**c**) Corresonding vascular density quantified from slit lamp images. For density quantification, n = 4 and asterisk represents p-value < 0.05 between suture OUT and IN. Error bars represent standard error of the mean (SEM). (**d,e**) Whole-mount dual immunofluorescence for CD31 and Collagen IV in suture OUT and suture IN, respectively. (**f**) Comparison between suture OUT and suture IN at both 24 and 120 h revealed delayed basement membrane deposition in suture OUT and continued active sprouting with suture IN.

Following nylon suture placement in the temporal rat cornea, a robust inflammatory and angiogenic response was induced. Four to five days after suture placement during the active angiogenic sprouting phase, new capillary sprouts invaded the cornea one-half to two-thirds of the distance from the limbal vessel arcade to the sutures. Sutures were removed (time 0 h) in the experimental suture OUT arm, whereas they were maintained in a parallel positive control arm (suture IN), Fig. 1a. Clinical slit lamp imaging revealed that suture removal did not halt sprout growth towards the sutures, which continued for 24 h (Fig. 1b). Significant reduction in vascular density of the new sprouts, however, was noted in both suture OUT and suture IN arms at 24 h (p < 0.001 and p = 0.003 respectively) relative to 0 h. This decline was more pronounced in the suture OUT arm at 72 h (p = 0.04) and 120 h (p = 0.004) relative to suture IN (Fig. 1c). Immunostaining of corneal tissue harvested at 24 h revealed that collagen IV-positive vascular basement membrane was absent in new sprouts 24 h after suture removal (Fig. 1d), whereas sprouts in the IN arm had nearly complete basement membrane coverage (Fig. 1e). By 120 h, substantial remodeling and regression was evident in the OUT arm as indicated by sparse vessels and empty basement membrane sleeves devoid of vascular endothelium. By contrast, in the IN arm vascular density was high, basement membrane persisted on many vessels (yellow; Fig. 1f) and new sprouting continued, indicated by the presence of CD31-positive sprouts without basement membrane (green; Fig. 1f).

### Acceleration of natural angiogenic remodeling suppresses granulocytes and enhances macrophage recruitment and vessel splitting

**Figure 2:**
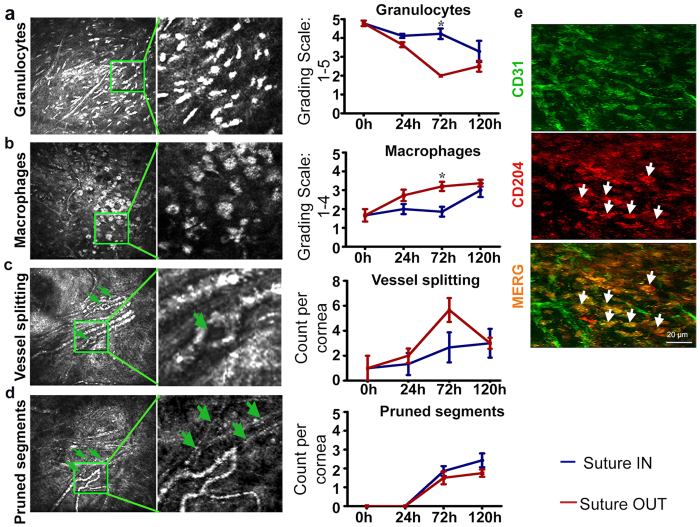
A shift from granulocyte to macrophages is enhanced by removal of the angiogenic stimulus. (**a**) Granulocyte cell infiltration; ANOVA p = 0.014 and p < 0.001 in suture IN and OUT arms respectively. (**b**) Macrophage infiltration; ANOVA p = 0.027 and p = 0.016 in suture IN and OUT arms respectively. (**c**) Vessel splits indicated by green arrowheads; ANOVA p = 0.007 in suture OUT. (**d**) Pruned vessel segments; ANOVA p < 0.001 in both suture IN and suture OUT, and the green arrowheads in D point to the pruned segments. For each micrograph the corresponding quantification is to the right, and for each measured parameter n = 4. Asterisks represent p < 0.05 between suture OUT and IN. Error bars represent SEM ± . (**e**) Cells positive for CD204 are indicated by white arrow heads.

By longitudinal *in vivo* confocal microscopy (IVCM) imaging in the rat corneas, inflammatory cell dynamics and capillary remodeling were investigated. In the suture IN arm from 0 h to 120 h, early infiltrating granulocytes were gradually reduced (p = 0.014), macrophages increased (p = 0.027), sprout tip abandonment increased (p < 0.001) and intraluminal vessel splits (intussusception) occasionally appeared (see Supplementary Movie) and (Fig. 2a–c).

In the suture OUT arm from 0 h to 120 h, reduction in granulocytes (p < 0.001), increase in macrophages (p = 0.016), increase in sprout tip pruning and abandonment (p < 0.001), and increased intussusception (p = 0.007) were noted. Granulocyte reduction and macrophage recruitment however, were stronger in suture OUT relative to suture IN (p = 0.01 and p = 0.007 respectively) (Fig. 2a,b), both at 72 h.

In both arms, vessel splitting peaked 72 h after suture removal (p = 0.04, relative to 0 h) (Fig. 2c), while sprout tip pruning (Fig. 2d) was equally elevated (p = 0.002) in both arms at 72 h relative to 0 h. Pruned sprout tips were non-perfused, confirmed by *in vivo* examination of flow characteristics and whole mount staining where CD31-negative basement membrane sleeves were noted. Infiltrating macrophages closely associated with regressing vessels expressed CD204 and CD31 (Fig. 2e).

As suture removal induced maximal morphologic changes at 72 h relative to the IN arm (Figs 1 and 2), it was hypothesized that these tissue-level changes would be regulated by genes expressed at an earlier time point. As a result, gene expression differences were investigated in both arms at 24 h. To do so, RNA was extracted from corneas of individual rats at 24 h and tested for sufficient concentration and good quality for further analysis, with RIN values ≥7 (see Supplementary Fig. S1).

### Differential gene expression analysis isolates genes more strongly modulated than VEGF-A

#### Analysis of differentially expressed genes (DEG)

**Figure 3:**
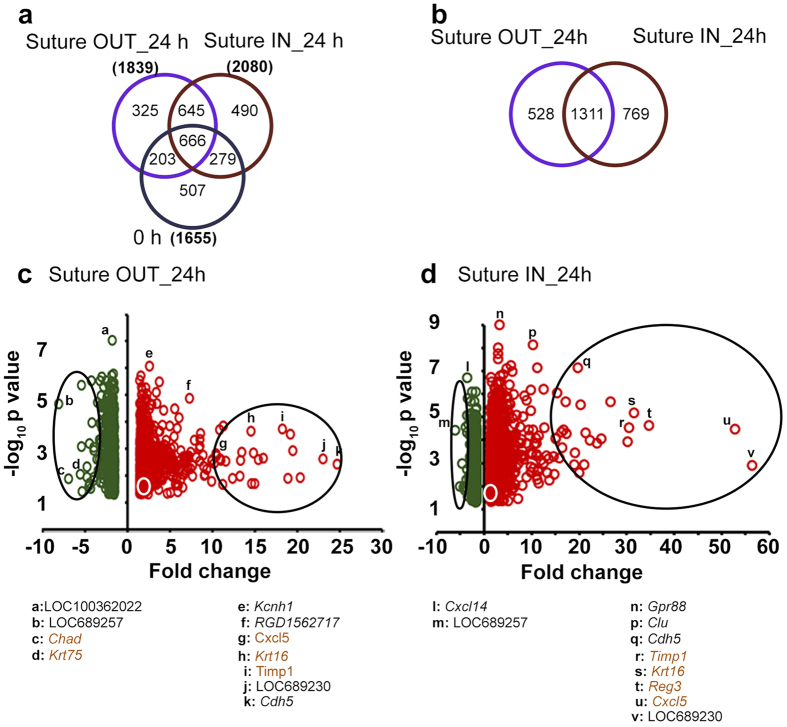
Differentially expressed genes (DEG) at 24 h suture IN and suture OUT. (**a**) Comparison of DEG at 0 h and 24 h. 0 h defines the starting point for the two arms. (**b**) Comparison of DEG between suture OUT and suture IN at 24 h only. (**c**) Volcano plot of the DEG in suture OUT and (**d**) Volcano plot of DEG in suture IN. In (**c,d**) the green and red colours represent down- and upregulated genes respectively and the white circles give the approximate location of *Vegfa*. Large black ovals highlight approximately the top 25 most up and 25 most down regulated genes. Specific genes are listed below. Those with significantly different FC between suture IN and OUT (p < 0.05) are highlighted in brown colour.

Rat whole genome microarray analysis yielded 1655 DEG genes at 0 h, and 1839 and 2080 DEG in suture OUT and IN respectively at 24 h, with all DEG having fold change (FC) p < 0.05 relative to non-sutured controls (Fig. 3a). Of the DEG in suture OUT and IN arms, 1311 genes were common (in terms of gene symbol) to both (Fig. 3b), with the majority of the DEG in the respective arms being up regulated (Fig. 3c,d).

#### Pathway enrichment analysis

**Figure 4:**
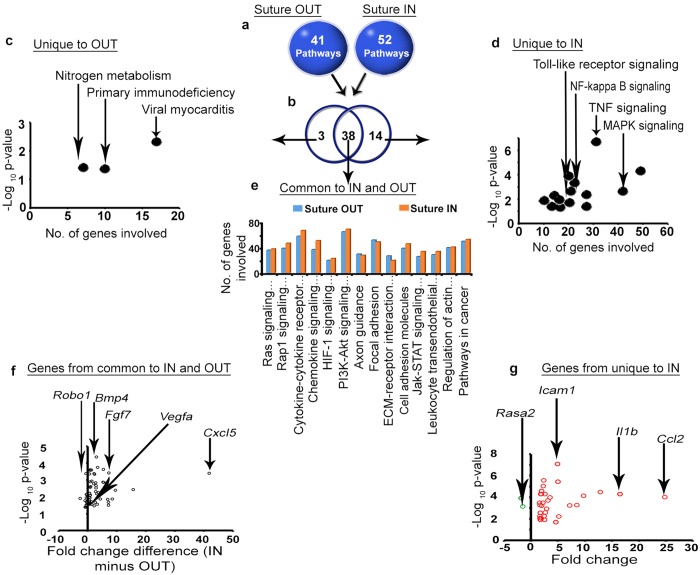
Pathways enriched in suture IN and OUT, and the genes involved in the selected pathways. (**a**) Number of pathways enriched in Suture OUT and IN arms. **(b)** Comparison of enriched pathways between suture IN and OUT. **(c)** Pathways uniquely enriched in the suture OUT arm. (**d**) Pathways uniquely enriched in the suture IN arm. (**e**) Pathways selected from those common to both arms. Pathway enrichment corrected p < 0.05 was considered significant. (**f**) Genes involved in the selected pathways common to suture OUT and suture IN. The x-axis indicates FC difference (IN minus OUT), and y-axis p-value (IN vs OUT). (**g**) Genes involved in the selected pathways uniquely enriched in the suture IN arm. The green and red colours in (**g**) represent down- and upregulated genes respectively. FC and p-value in (**g**) are relative to the native control cornea.

Classification of the above DEG genes into pathways at 24 h yielded 41 and 52 significantly enriched Kyoto Encyclopedia of Gene and Genome (KEGG) pathways in suture OUT and suture IN arms respectively (Fig. 4a). Comparison of enriched pathways between arms resulted in 38 pathways commonly enriched in both arms, 3 pathways uniquely enriched in 24 h suture OUT, and 14 uniquely enriched in suture IN (Fig. 4b). A detailed list of the pathways is provided (see Supplementary Table S1). Pathway classification analysis revealed nitrogen metabolism among others uniquely enriched in suture OUT (Fig. 4c) while MAPK, TNF and NF-kappa signalling were uniquely enriched in suture IN (Fig. 4d). Signal transduction associated pathways with a link to inflammation/immunity and angiogenesis were commonly enriched in suture IN and suture OUT (Fig. 4e). Genes involved in the selected pathways were retained if FC (suture IN vs Suture OUT) had p < 0.05. Genes in (Fig. 4f) were identified from the common pool of pathways, while genes presented in (Fig. 4g) were identified from pathways unique to IN. Selection of pathways for downstream analysis was based on the KEGG pathway maps and classification criteria as a guide (available at http://www.genome.jp/kegg/pathway.html).

A summary of genes involved in the analysed pathways above was generated, duplicate gene ID’s were removed and only genes with significant FC difference between IN and OUT arms were retained. Supplementary Table S2 gives the top 25 genes sorted by p-value, descending, and ascending FC difference (IN vs OUT).

#### Biological process enrichment analysis

**Figure 5:**
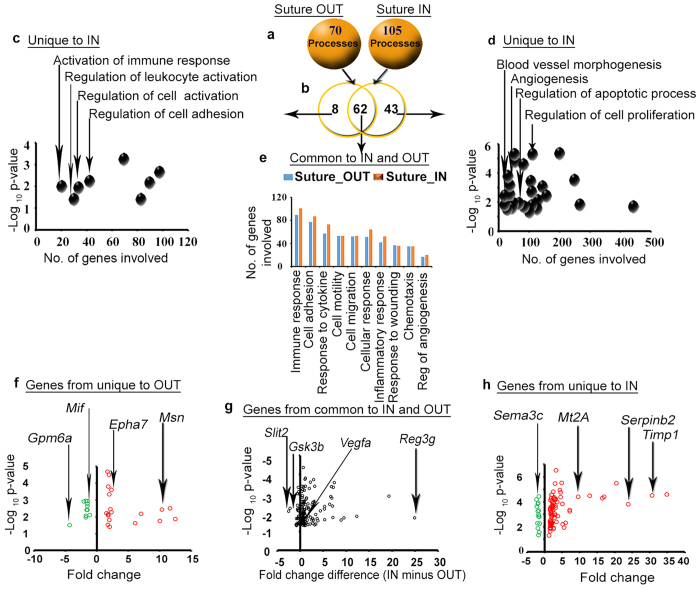
Biological processes enriched in suture IN and OUT, and the genes involved in the selected processes. (**a**) Number of biological processes enriched in Suture OUT and IN arms. (**b**) Comparison of the biological processes between suture OUT and suture IN. (**c**) Biological processes uniquely enriched in the suture OUT. (**d)** Biological processes uniquely enriched in the suture IN. (**e**) Biological processes commonly enriched in both suture OUT and IN. Biological process enrichment was corrected by limiting to those processes with p < 0.05. (**f**) Genes involved in the selected biological processes uniquely enriched in suture OUT. (**g**) Genes involved in the selected biological processes common to suture OUT and suture IN. The x-axis indicates FC difference (IN minus OUT) and y-axis is p-value (IN vs OUT). (**h**) Genes involved in the selected biological processes uniquely enriched in suture IN. In f and h, FC and p-values are relative to the native control cornea, and green and red represent down- and upregulated genes respectively.

In addition to pathways, the DEG at 24 h were classified into biological processes. 70 and 105 biological processes were enriched in suture OUT and IN, respectively (Fig. 5a). 62 processes were commonly enriched, 8 uniquely enriched in suture OUT and 43 uniquely enriched in suture IN (Fig. 5b). Supplementary Table S3 provides a list of the enriched biological processes. Processes such as regulation of cell activation and cell adhesion were unique to suture OUT (Fig. 5c), while regulation of apoptosis, regulation of cell proliferation, and blood vessel morphogenesis among others were unique to suture IN (Fig. 5d). Inflammatory response, regulation of angiogenesis and immune response were among those processes commonly enriched in both arms (Fig. 5e). Genes associated with processes unique to suture OUT, IN, and common processes are presented in Fig. 5f–h.

For comparison purposes, biological process overrepresentation by Cytoscape BiNGO software generated biological processes similar to those obtained from STRING analysis (Supplementary Table S3b). Finally, a summary of genes involved in the selected and analysed biological processes from STRING analysis was generated, duplicate genes were removed and only genes significantly different in fold change between suture IN vs Suture OUT were retained. A similar sorting criteria as described for the pathways was repeated (see Supplementary Table S4). Only the top most 25 genes are shown. Comparing pathway enrichment and biological process enrichment analysis, 69 genes were similarly identified from both analysis approaches, 56 genes were uniquely identified from the pathway analysis, and 118 genes were uniquely identified from the biological process enrichment analysis (Supplementary Fig. S5).

To summarise the whole transcriptome data analysis, the gene lists from both pathway and biological process analysis were pooled and duplicate genes were removed. With reference to published literature, the genes were grouped into proangiogenic/proinflammatory (genes downregulated with suture removal) and pro-remodeling/inhibitory (upregulation with suture removal) gene lists. Relative to the magnitude of VEGFA up-regulation after suture placement and down-regulation 24 h after suture removal, a range of proangiogenic/proinflammatory genes had substantially higher magnitudes of up- and down-regulation (Table 1).

### Confirmation of regulation of selected VEGF-independent factors by qPCR and immunohistochemistry staining

**Figure 6:**
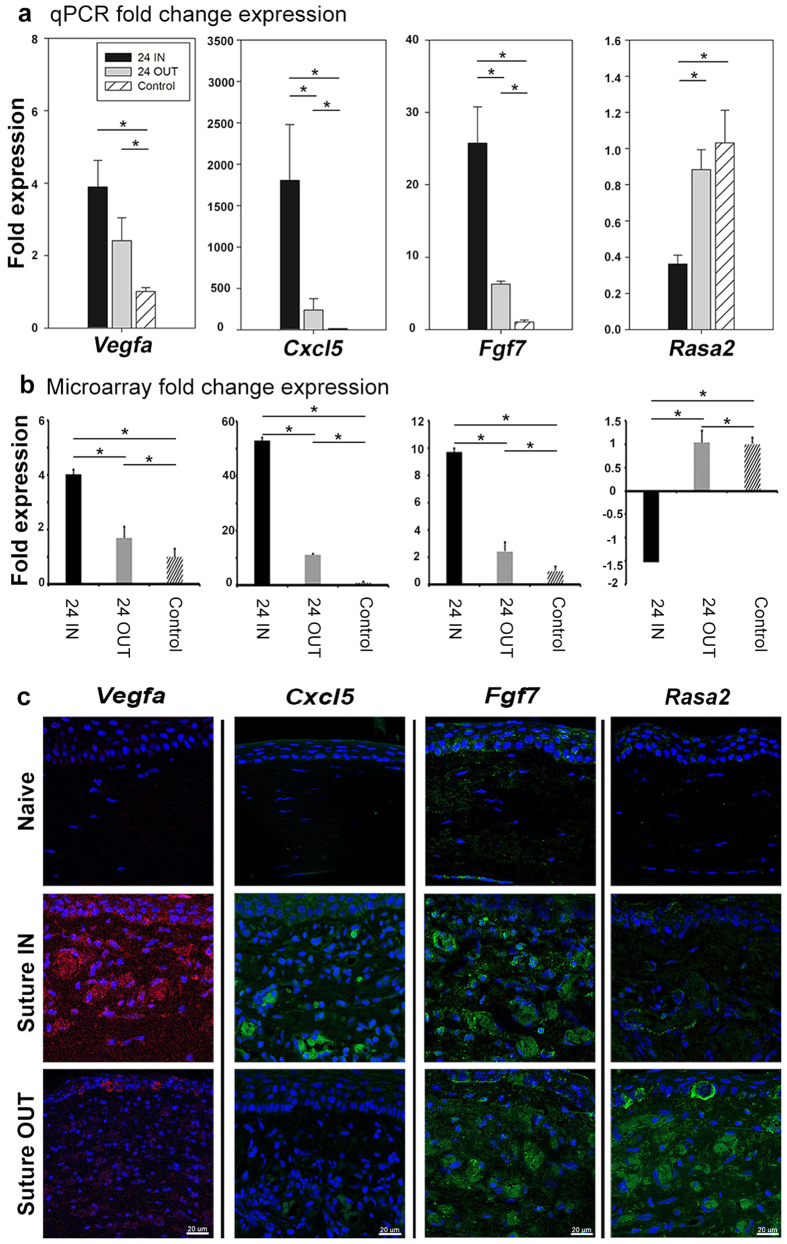
Confirmation of regulation of selected genes at 24 suture IN and OUT, by qPCR and immunohistochemistry. Gene expression confirmation by qPCR, and protein level expression by immunohistochemistry. Panel (**a**) indicates gene expression by qPCR, (**b**) indicates the microarray gene expression and (**c**) is the protein expression in the tissue for *Vegfa*, *Cxcl5*, *Fgf7* and *Rasa2*, respectively. For qPCR, ANOVA p-values were 0.006, <0.001, <0.001 and 0.009, for *Vegfa*, *Cxcl5*, *Fgf7* and *Rasa2*, respectively. Pairwise significance between groups with p < 0.05 is indicated by asterisks. For microarray, p-values between suture IN and suture OUT were 4.49E-04, 3.89E-04, 3.66E-04 and 8.56E-03, for *Vegfa*, *Cxcl5*, *Fgf7* and *Rasa2*, respectively. The asterisks represent a significant difference p < 0.05 between groups. Error bars for qPCR and microarray represent standard deviation. Microarray data represents 4 corneas/group and qPCR data 3 corneas/group with 2 technical replicates/sample.

From the sub grouping in Table 1 representative genes were selected for qPCR and immunohistochemical staining validation of fold change expression: *Vegfa* (as control), *Fgf7* and *Cxcl5* (as proangiogenic and proinflammatory), and *Rasa2* (as inhibitory/remodeling). The fold change expression of these genes by qPCR (Fig. 6a) Indicated a direction and relative magnitude in agreement with the microarray data (Fig. 6b). In particular, significant reduction in *Cxcl5* (1588.9 ± 0.8 to 194.6 ± 1.3, p < 0.001) and *Fgf7* (24.7 ± 0.5 to 6.3 ± 0.1, p < 0.001), and increase in *Rasa2* (0.3 ± 0.3 to 0.9 ± 0.2, p = 0.009) occurred with suture removal. By immunohistochemistry (Fig. 6c), expression of *Rasa2* at the protein level increased following suture removal relative to the suture IN arm. The expression of *Fgf7*, *Cxcl5* and *Vegfa* all decreased following suture removal relative to Suture IN. Protein expression of *Rasa2* and *Fgf7* was localized to the vessel walls and lumens, *Cxcl5* localized to epithelium and the vessel lumen, and *Vegfa* localized to epithelium, vessel walls and lumens. The expression profile of the assayed factors at the protein level confirmed the trend of qPCR microarray results.

**Figure 7:**
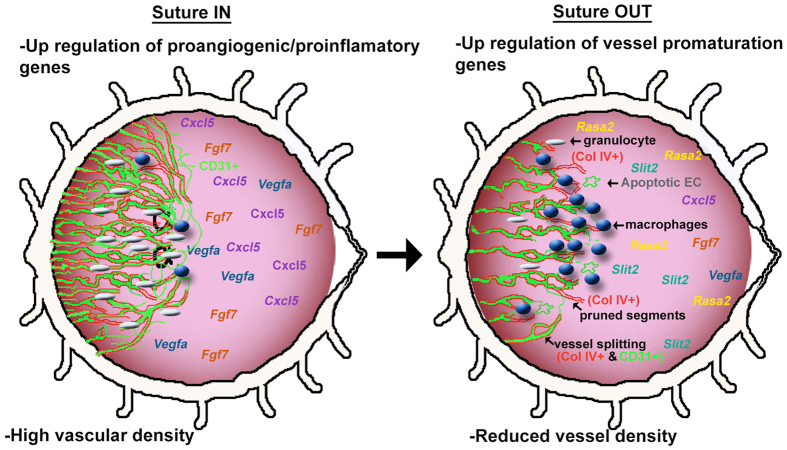
Conceptual illustration of corneal remodelling upon reversal of an angiogenic stimulus. The diagram illustrates conceptually the changes in the corneal tissue that are initiated with suture removal, both at the tissue phenotypic level and at the gene and protein expression level.

In this study, blood vessel regression was induced by removal of the angiogenic stimulus during an active sprouting phase and 24 h later, a chain of events was initiated that included a suppression of granulocytes, a build-up of remodeling-phenotype macrophages, a build-up of pruned segments (Coll IV^+^ basement membrane without vascular endothelium), vessel splitting, reduced expression of proinflammatory and proangiogenic genes, and an upregulation of inhibitory and putatively pro-remodeling genes. Overall, there was a decrease in inflammation and blood vessel density. The results of the study have been summarized in a conceptual diagram (Fig. 7).

## Discussion

Inflammation plays a central role in the corneal suture model, mimicking many pathologic situations. In the pathologic pro-angiogenic milieu, granulocytes were abundant and new sprouting occurred while remodeling was limited and remodeling-type macrophages were suppressed. Upon suture removal, rapid resolution of inflammation and restoration of corneal transparency was characterized by strong downregulation of inflammatory and proangiogenic factors triggering a remodeling response by granulocyte suppression, M2-phenotype macrophage recruitment, endothelial cell degradation, and enhanced vessel splitting. At the tissue level, resolution of edema and reduction of sprout density were clinically apparent Fig. 7.

Macrophages have remarkable plasticity and are reported to be important for the induction of regression of lens vasculature during development, and in inhibiting the growth of abnormal blood vessels in the eye in AMD^31^,^32^,^33^,^34^. In tumours, tumour-associated macrophages positive for CD204 were considered to promote tumour growth^35^. In our model, we observed a build-up of CD204-positive macrophages following suture removal. These macrophages could be responsible for the reduction in inflammation and edema, and normalization of vasculature which we noted. These findings are in line with the observation that M2 macrophages in the injured retinal vasculature promoted tissue remodelling and stabilization by modulating the inflammatory response, reducing oxidative stress and apoptosis and promoting tissue repair^36^.

Rat whole transcriptome analysis identified a number of candidate genes directly implicated in these events. As expected, *Vegfa* expression was upregulated during sprouting and was suppressed upon suture removal (FC difference 2.32). The magnitude of upregulation of *Vegfa* with suture placement and downregulation with suture removal, however, was only moderate and was surpassed by many other genes. Expression of chemokine ligands and receptors such as *Cxcl1*, *Cxcl3*, *Cxcl5*, *Ccl2*, *Ccl7*, and *Cxcr2* was especially strong, as was their subsequent suppression. These factors are known angiogenic and inflammatory chemokines, responsible for granulocyte/neutrophil invasion and trafficking (*Cxcl1, Cxcl3, Cxcl5* and their common receptor *Cxcr2*), and monocyte invasion (*Ccl2, Ccl7*)^37^,^38^,^39^,^40^. Significant suppression of *Csf-3r* and *Csf-2rβ* was also noted, which are attractants of proangiogenic granulocyte and macrophage populations^41^. Suppression of the expression of several interleukins and receptors was also found, including *IL1β, IL1-R2, IL1-RL1* (receptor for *IL-33*), *IL18-RAP*, and *IL-24.* All of these factors are considered inflammatory and/or pro-angiogenic (with the exception of *IL-24* which is considered to be an inhibitor of angiogenesis)^42^,^43^,^44^,^45^,^46^. The observed reduction in granulocytes starting at 24 h and peaking at 72 h was likely mediated by suppression of these chemokines, colony stimulating factors and interleukins.

The inflammatory cytokine *Tgfβ1* is a potent chemoattractant of myeloid cells^47^ and *Bmp-4* is a member of the Tgfβ superfamily that can activate MAPK signaling pathways^48^, whose suppression upon suture removal concurs with a reduction of inflammation. The specific kinases *Map4k4* and *Map2k*6 were moderately upregulated upon suture removal, while moesin (which *Map4k4* phosphorylates) was significantly overexpressed and was downregulated upon suture removal, indicating an impairment of EC migration^49^. *Tgfβ1*, however, is also considered to promote vessel maturation by stimulating production of ECM and promoting smooth muscle cell development^50^,^51^; however, it is also expressed in vascular ECs where it can be either pro- or antiagngioenic, depending on the concentration and context^52^. For example, the Tgfβ1-Alk5 signaling pathway induces Id1 expression which stimulates EC migration and proliferation, while the same pathway induces the plasminogen activator inhibitor Pai1 in ECs which prevents degradation of the provisional matrix around new vessels^53^. PAI1 and PAI2 have also been associated with smooth muscle cell survival^54^. In our model, the expression of *Pai1* (*Serpine1*), *Pai2* (*Serpinb2*), *Id1* and *Bmp4* all decreased significantly upon suture removal, indicating resolution of inflammation, inhibition of EC migration and proliferation, and vessel degradation functions for these genes in the present context.

Several enzymes also likely to play key roles in supporting inflammation and angiogenesis in the present model. Several members of the dual-specificity phosphatase (Dusp) family of genes (*Dusp1, Dusp6, Dusp7*) were suppressed upon suture removal. Dusp family genes are known as important modulators of MAPK signaling^55^, although their mechanism of action in the present model remains to be elucidated. Targeting of Dusp family genes, however, has attracted much interest in recent years as this group of phosphatases has broad-ranging activity in many human diseases and represents a potential therapeutic target^56^. Additionally, matrix metalloproteinases (MMPs) and their inhibitors, notably TIMPs, have also been shown to have an important role in inflammation and angiogenesis^57^. *Mmp9*, secreted by neutrophils^58^ was upregulated with suturing but dramatically suppressed upon suture removal. Mmp9 is considered to represent a trigger enzyme for promoting angiogenesis^59^. Timp1, an inhibitor of Mmps, is generally considered to be an inhibitor of angiogenesis^57^ but in the present study was overexpressed at 0 h, and highly suppressed with suture removal, indicating a potential inflammatory and/or angiogenic role in our model. Mmp9 is also critical for producing potent endogenous angiogenesis inhibitors such as endodstatin and angiostatin by proteolysis of collagen and fibronectin present in the extracellular matrix^60^, and inhibition of Mmp9 has not led to clinically meaningful reduction in tumor angiogenesis and growth^61^. The specific role of Mmp9 and Timp1 in inflammation-induced corneal neovascularization requires further investigation.

Fibroblast growth factors (FGFs) are proangiogenic factors also involved in wound healing^62^. *Fgf7* (also known as keratinocyte growth factor or KGF) was highly upregulated after suturing and strongly suppressed after suture removal. *Fgf7* has previously been shown to be proangiogenic in the rat cornea and can activate MAPK signaling^63^. Moreover, *FGF7* has been shown to stimulate *VEGFA* in colorectal cancer tumor cells^64^ and *FGF7* expression enhances cell adhesion to collagen IV of the vascular basement membrane^65^. The strong suppression of *Fgf7* in our model may have promoted EC detachment and abandonment of sprout tips in our model, leaving the collagen IV-positive empty basement membrane sleeves observed 120 h after suture removal.

In addition to the above mentioned genes, several other putatively inflammatory and/or angiogenic factors were identified with strong upregulation in the IN arm and significant suppression upon suture removal. These factors included *Reg3g*, *Krt16*, *Serpinb2, S100a8, Rarres2, Serpine1, Actn1, Icam1, Lamc2*, *Fos*, *Mt2A*, *Niacr1*, *Fam110c, Egr1, Nov,* and *Trem1*^66^. All were upregulated with suturing and suppressed following suture removal to a greater degree than *VegfA*. Most of these genes are known to have proangiogenic, tumorigenic, cell migration and proliferation effects, and several are novel in the context of the eye. The strong expression of these genes makes them possible candidate targets for suppressing inflammation and angiogenesis independently of *Vegf*.

Overwhelmingly, suture removal suppressed inflammatory and angiogenic factors by large magnitude fold changes in gene expression, while inhibitory and putative remodeling factors were upregulated by a comparatively modest amount (Table 1). This suggests that suppression of the inflammatory and pro-angiogenic environment is important for remodeling and regression to occur. It also suggests that either the strong downregulation of inflammatory and angiogenic factors may itself trigger microvascular remodeling, or that the putative anti-angiogenic and pro-remodeling factors are very potent such that only a small degree of upregulation is sufficient to effect remodeling and regression. The interplay between suppression of promoters and expression of inhibitors, however, requires further investigation.

Several upregulated factors are of potential importance. *Fgf13* or fibroblast homologous factor 2 (FHF-2) has been reported to promote resistance to cancer therapeutics^67^ and *NOTCH2* expression has been shown to be a negative regulator of angiogenesis^68^. Other genes significantly upregulated upon suture removal were *Slit2, Robo1, Rasa2, Gsk3b, Lama2, Epha7, Sema3a/c, CD36, Phlpp2, Magi3, Syne2,* and *Plxna4a*. Some of these are known inhibitors of angiogenesis (e.g. *Slit/Robo, CD36, Rasa2, Gsk3b, Sema3a/c, Phlpp2, Magi3, Epha7*)^69^. Others regulate endothelial cell shape and reorganization (*Syne2*), or axonal guidance (*Sema3a/c, Slit2, Robo1, Lama2, Plxna4a*). Slits particularly have been reported to exhibit both pro- and anti-angiogenic functions. In a recent study using conditional knockout mice, *Slit2* was shown to promote angiogenesis through interaction with *Robo1* and *Robo2* receptors in mouse postnatal retina^70^. Another study indicated an anti-angiogenic role of *Slit2* interaction with *Robo1* and *Robo 4* in corneal angiogenesis^69^.

One of the most upregulated genes upon suture removal was RAS p21 protein activator 2 (Rasa2). Rasa2 is an inhibitory regulator of the Ras-cyclic AMP pathway, and binds inositol tetrakisphosphate (IP4) and phospholipid. Rasa2 has recently been shown to have tumor suppression activity^71^, and from our analysis with STRING (http://string-db.org/), we found that Rasa2 is predicted to both activate and inhibit Rras protein among others. Rras is said to regulate the organisation of actin cytoskeleton to influence vascular barrier integrity^72^. Sawada *et al*., recently reported that Rras controls the response of endothelial cells to VEGF, suggesting an underlying mechanism by which Rras regulates angiogenesis^73^. Using mice, the ablation of Rras enhanced angiogenesis in tumor implants^74^. Conversely, the gain of function of Rras in ECs improved the structure and function of VEGF-induced blood vessels in Matrigel implants^75^, indicating a function for Rras in normalizing pathological vasculature.

Additionally, several of the above genes have not previously been recognized and represent potential targets for future studies.

Finally, some genes with strong expression in our model behaved in a counter-intuitive manner, such as *Socs3* and *IL-24*. These are known inhibitors of inflammation/angiogenesis that were significantly upregulated in the active sprouting phase and downregulated upon suture removal, and further investigation of these factors is required.

The cornea being naturally avascular provides an opportunity to easily image, measure and quantify angiogenesis *in vivo*, and allows for investigation of how the cornea regains its avascularity following removal of the angiogenic stimulus. Cornea models have been used extensively in tumour biology^76^,^77^ despite the differences in tumor environments and the avascular cornea. The genes identified in the present study are therefore relevant in the context of the cornea; however, they may also represent targets in other vascularized tissues and in the tumor microenvironment, but further study of the identified genes in these other contexts is required.

The results of this study underscore the importance and redundancy of multiple pathways in the present model of angiogenesis, and the model is primarily relevant only for the cornea which has its own mechanism for restoring avascularity that may be different from other tissues. Nevertheless the cornea model may reveal new factors that could have therapeutic potential even in other tissues. Interestingly, many VEGF-independent factors were modulated in the context of endogenous restoration of corneal avascularity, and to a greater degree than *VegfA*. In addition to the identification of possible gene targets that were confirmed at the protein level in the corneal tissue, the results of this study suggest several means to influence vessel remodeling, such as targeting inflammatory chemokines/cytokines alone or in combination with anti-VEGF treatment, promoting overexpression of endogenous inhibitory and remodeling factors, or a combination of such approaches. Modulating the remodeling of pathologic angiogenic vasculature could promote a stable, noninvasive, non-leaky and non-damaging vascular phenotype to prevent blinding eye pathology, while providing a means to improve anti-angiogenic drug delivery and mitigate drug resistance in the eye and beyond.

## Material and Methods

### Experimental model, maintenance and ethics statement

Pathogen-free male Wistar rats, aged 12–16 weeks and weighing 350–400 g (Scanbur AB, Sollentuna, Sweden) were housed at the Linköping University animal department in an environment controlled by a Heating, Ventilating and Air conditioning system. Standard dark: light cycle of 12:12 hours was maintained, noise levels were maintained below 85 dB. Standard rodent food and water was at ad libitum. A two weeks quarantine before experimentation was maintained. Suture-induced inflammatory corneal neovascularization model was used^78^. Experiments were in accordance with guidelines of the Regional ethics committee for Animal Experiments at Linköping University, Sweden and in line with the Association for Research in Vision and Ophthalmology (ARVO) guidelines for the Use of Animals in Ophthalmic and Vision Research. All animal experimental protocols were approved by the Linköping Regional Animal Ethics Committee (Approval no. 7–13) prior to the start of the study.

### Study design and experimental procedure

Two 10-0 nylon sutures were placed temporally in the cornea of the right eye at a distance of 1.5 mm from the pre-existing limbal vessels (see Supplementary Fig. S2). ‘0 h’ time point was when neovessels had reached one-half to two-thirds of the distance between pre-existing limbal vessels and the sutures. The 0 h time point typically corresponded to day 4 after suture placement. This was taken to represent an active sprouting phase with little vessel remodeling or maturation. The rats were then divided into two arms, ‘suture OUT’ in which sutures were removed from the cornea at 0 h time point and ‘suture IN’ where sutures were left in place. Rats were then randomly assigned to 24, 72 and 120 h sampling time points for each arm (Fig. 1a). Rats with non-sutured (naïve) corneas served as controls. Experiments for both arms were run concurrently, and for each sampling time point including control, n = 5 rats.

### Slip lamp imaging

A drop of tropicamide 0.5% (5 mg/ml) was administered into the sutured eye to dilate the pupil. Examination was performed using a Micron III rodent slit lamp camera (Phoenix Research Laboratories, USA). Vascular density was measured by determining the mean pixel percentage representing vessels in three 150 × 150 pixel sub-regions selected from slit lamp images, and n = 4/time point.

### *In vivo* confocal microscopy imaging

Images acquired by IVCM^79^ were analysed for inflammatory cell infiltration, intraluminal holes/splitting and sprout tip pruning/abandonment. Semi-quantitative grading of inflammatory cells was used (Supplementary Fig. S3; granulocytes, and Supplementary Fig. S4; macrophages), while intraluminal holes and sprout tips were counted manually. Three representative images per feature were selected at each time point. Values were averaged across observers and for all rats at a given time point.

### Whole mount immunostaining and fluorescent imaging

Samples were treated with cold acetone for 30 mins, washed in PBS-A 3 × 20 mins and blocked with normal donkey serum (1:20) for 2 hrs at rt, and after, stained overnight (O/N) at 4 °C with anti-collagen IV antibody (rabbit polyclonal to collagen IV, Abcam 19808). In the dark, samples were washed 3 × 30 mins PBS-A, and stained for 2 hrs with a DyLite 649 donkey anti-rabbit antibody (1:200) at rt. Next, was a 3 × 30 mins wash with PBS-A, then blocked with normal donkey serum for 2 hrs at rt, and an O/N staining at 4 °C with anti-CD31 antibody (TLD-3A12) (mouse monoclonal to CD31 Abcam 64543). Samples were washed 3 × 30 mins PBS-A followed by a 2 hrs at rt staining with donkey anti-mouse antibody (secondary) (1:250), and washed again 3 × 30 mins PBS-A, then mounted with fluorescent mounting medium (Dako S 3023). Images were taken using a laser-scanning confocal fluorescence microscope (Zeiss LSM 700) at 40x.

### RNA isolation and quality check

Cornea tissue excluding the scleral rim, and ≈30 mg was disrupted using a hand held tissueRuptor and disposable Probe (Qiagen, Hilden, Germany) in a 2 ml tube. Total RNA was extracted from the lysate using RNeasy Mini Kit (Qiagen, Hilden, Germany) without DNase treatment. RNA was eluted in 50 μl of RNase free H_2_O and quantified using Nano drop 2000 (Thermo scientific). Speed vac was used to concentrate samples when needed. RNA integrity was determined using an Agilent 2100 Bio analyser (Agilent Technologies Inc., Paolo Alto, CA, USA) and RNA integrity number (RIN) of ≥7 was the cut-off for sample inclusion for downstream analysis.

### Target preparation and hybridisation

Gene expression analysis was performed for both suture IN and suture OUT at 24 h using GeneChip Gene 2.0 ST 100-Fornat Array (Affymetrix Inc). A total of 16 RaGene-2.0-ST microarrays were run to correspond to 4 chips per time point (i.e. Control, 0 h, 24 h suture IN, and 24 h suture OUT). An input of 100 ng of total RNA was used for target preparation following the manufactures instructions (GeneChip^®^ WT PLUS Reagent Kit, P/N 703174 Rev. 2, Affymetrix Inc).

### Identification of differentially expressed genes

DEG at each time point were obtained using filters; p-value < 0.05 and FC ≤ −1.5 or ≥1.5, relative to the control. The q-value of the DEG was also taken into account, and used for selecting the final gene list for FC validation.

### Pathway enrichment analysis

DEG at the 24 h (suture IN and suture OUT), both up and down regulated genes were used for Encyclopedia of Gene and Genome (KEGG) pathway enrichment using Search Tool for the Retrieval of Interacting Genes/Proteins (STRING) software v.10 (http://string-db.org/).The p-value, p-value_fdr^80^ and p-value_bonferroni^81^ were set at p < 0.05. The significantly enriched pathways were compared between arms using Venny 2.1 (http://bioinfogp.cnb.csic.es/tools/venny/). From the resultant pathway subgroupings i.e. ‘’uniquely enriched-suture OUT’, ‘‘uniquely enriched-suture IN” and ‘‘common to suture IN and suture OUT”, representative pathways were selected and the genes involved in them were identified. The genes were compared between the two arms and only genes with p < 0.05 (FC suture IN vs suture OUT) were of “interest”. The gene list was further scrutinised based on q < 0.05 in each arm relative to the control. Genes with q < 0.05 were eligible for qPCR validation of FC.

### Biological process enrichment analysis

An analysis approach similar to that of pathway enrichment analysis described above was repeated for biological process enrichment. In addition to STRING, Cytoscape BiNGO-overrepresentation analysis was also performed. BiNGO overrepresentation was obtained using a Hypergeometric test, and Benjamini & Hochberg False Discovery Rate (FDR) correction^80^, with the significance level set at 0.05, and testing was done using the whole annotation as a reference set. The significantly enriched and overrepresented biological processes were compared between the two arms in the same way as was done for pathway comparison. A flow chart summarising the analysis approach for both pathway and biological processes is shown in Supplementary Fig. S5.

### Validation of microarray data by quantitative real-time PCR (qPCR)

RNA was extracted from cornea tissue, three independent samples/time point. At least 653 ng of cDNA was synthesized using SuperScript VILO cDNA Synthesis Kit (Invitrogen) in accordance with the instructions from the manufacturer. Then 653 ng of cDNA was used for qPCR with primers for rat-specific *Gapdh*, *Vegfa*, *Fgf7*, *Rasa2* (TaqMan, Applied Biosystems) and *Cxcl5* (PrimeTime, Integrated DNA Technologies). TaqMan Fast Advanced Master Mix (Applied Biosystems) was used for all the above primers, in accordance with manufacturer guidelines. Two technical replicates per sample, and three biological replicates per time point, per treatment were used. Threshold cycle (Ct) values were normalised to *Gapdh*, and gene expression measured by relative quantitation method.

### Immunohistochemical staining for *Vegfa*, *Cxcl5, Fgf7* and *Rasa2*

The vascularised area of the cornea in both study groups was carefully dissected out immediately after animal sacrifice. Non sutured corneas of approximate size was used as the control. Tissues were fixed with 4% formaldehyde (Histolab, Gothenburg, Sweden) and processed for paraffin embedding, and sagittal sections 5-μm thick were prepared for Immunohistochemical staining with primary antibodies for *Fgf7* (orb107478), *Rasa2* (orb254207), *Cxcl5* (orb13450) (1:200; Biorbyt, CA, San Francisco, USA), and visualised with DyLight 488 (1:500; Thermo Fisher Scientific, Waltham, MA, USA). Primary antibody for *Vegfa* (1:100; GeneTex, Simpson, PA, United States) (GTX21316) was visualised using DyLight 519 (1:500; Thermo Fisher Scientific, Waltham, MA, USA). Stained sections were mounted with ProLong Gold antifade regent (Invitrogen, Thermo Fisher Scientific, Waltham, MA, USA) and imaged. Immunohistochemistry was performed to analyse protein expression and localisation of the targets in the cornea.

### Statistical analysis

Both slit lamp and IVCM data was analysed using GraphPad Prism 5 (GraphPad Software, Inc. CA 92037 USA). One-way analysis of variance (ANOVA) using Turkey’s multiple comparison Test was used for multiple comparisons across groups when variances were equal and the data normally distributed. A two tailed p-value < 0.05 was considered significant. For data not normally distributed, Mann-Whitney U test was used.

For microarray data, a p-value < 0.05 was considered significant, and FC ≤ −1.5 or ≥1.5 = differentially expressed. Corrected p-value (q-value) for each probe was also calculated, and q < 0.05 was considered significant. STRING p-value, p-value_fdr and p-value_bonferroni were all set < 0.05. Using Cystoscope BiNGO, significant overrepresentation was obtained using a Hypergeometric test, and Benjamini & Hochberg False Discovery Rate (FDR) correction^80^ with the significance level set at 0.05.

For qPCR, FC across groups was compared using ANOVA, using the Holm-Sidak multiple comparison method to isolate pairwise differences. A two-tailed alpha level of <0.05 was considered significant. SigmaStat 3.5 for Windows (Systat Software Inc., Chicago, USA) was used for analysis.

## Additional Information

**How to cite this article**: Mukwaya, A. *et al*. Factors regulating capillary remodeling in a reversible model of inflammatory corneal angiogenesis. *Sci. Rep.* **6**, 32137; doi: 10.1038/srep32137 (2016).

## Supplementary Material

Supplementary Information

Supplementary Movie S1

## Figures and Tables

**Table 1:** Summary of genes of interest from pathway and biological process analysis.

Symbol	FC difference IN-OUT
A. Proinflammatory/proangiogenic genes	
*Cxcl5*	41.67
*Reg3g*	24.84
*Krt16*	19.28
*Ccl2*	15.6
*Serpinb2*	15.18
*Timp-1*	12.29
*Il1b*	9.46
*S100a8*	8.82
*Cxcr2*	8.46
*Fgf7*	7.25
*Csf3r*	7.04
*Mt2a*	6.97
*Rarres2*	6.86
*Mmp9*	6.78
*Msn*	6.59
*IL-1r2*	6.39
*Serpine1*	5.41
*Socs3*	5.3
*Ccl7*	5.25
*IL-1rl1*	5.19
*Egr1*	4.64
*IL-24*	4.32
*Cxcl1*	3.78
*IL-18rrap*	3.61
*Nov*	3.32
*Lamc2*	3.32
*Trem1*	3.08
*Csf2rb*	3.07
*Bmp-4*	2.96
*Nos2*	2.81
*Otub1*	2.74
*Actn1*	2.73
*Fam110c*	2.68
*VegfA*	2.32
*Niacr1*	2.3
*Cxcl3*	2.14
*Dusp6*	1.76
*Angptl4*	1.59
*Myc*	1.29
*Sema7a*	1.01
*Id-1*	0.88
*Spred3*	0.82
*Spry4*	0.79
*Wnt7b*	0.63
B. Pro-remodeling genes	
*Cyld*	−2.82
*Slit2*	−2.56
*Rasa2*	−2.55
*Gsk3b*	−2.23
*Lama2*	−0.93
*Syne2*	−0.9
*Epha7*	−0.85
*Sema3c*	−0.84
*Ptprd*	−0.74
*Notch2*	−0.67
*Cdc42bpa*	−0.59
*Cd36*	−0.56
*Plxna4a*	−0.55
*Sorbs1*	−0.53
*Phlpp2*	−0.51
*Robo1*	−0.48
*Fgf13*	−0.42
*Magi3*	−0.26
*Map4k4*	−0.24
