# Supplementary material for: Factors regulating capillary remodeling in a reversible model of inflammatory corneal angiogenesis
Source: Sci Rep. 2016 Aug 26;6:32137. doi: 10.1038/srep32137 (PMC4999823; doi:10.1038/srep32137)
Supplement: Supplementary Information [file srep32137-s1.doc]

**Factors regulating capillary remodeling in a reversible model of inflammatory corneal angiogenesis**

Anthony Mukwaya^1^, Beatrice Peebo^1^, Maria Xeroudaki^1^, Zaheer Ali^2^, Anton Lennikov^1^, Lasse Jensen^2^, and Neil Lagali^1*^

^1^Department of Ophthalmology, Institute for Clinical and Experimental Medicine, Faculty of Health Sciences, Linkoping University, 58183 Linköping, Sweden

^2^Department of Medical and Health Sciences, Division of Cardiovascular Medicine, Linköping University, 581 83 Linköping, Sweden

*Corresponding author

Email address: [neil.lagali@liu.se](mailto:neil.lagali@liu.se)

**SUPPLEMENTARY INFORMATION**

**Supplementary movie S1:** Vessel splitting in single capillaries as observed by *in vivo* confocal microscopy in the rat cornea.

**Pathway enrichment analysis.** From STRING pathway enrichment analysis, only pathways satisfying multiple testing with p-value < 0.05 were considered for selection and for an in-depth downstream analysis.

**Supplementary Table S1**. List of selected pathways in Suture IN, intersection and in Suture OUT. Shown is the GO_id number and the pathway term.

| **Pathways uniquely enriched in Suture OUT (3)** | |
| --- | --- |
| **GO_id** | **Term** |
| 910 | Nitrogen metabolism |
| 5340 | Primary immunodeficiency |
| 5416 | Viral myocarditis |
|  |  |
| **Pathways uniquely enriched in Suture IN (4)** | |
| **GO_id** | **Term** |
| 4010 | MAPK signaling pathway |
| 4064 | NF-kappa B signaling pathway |
| 4620 | Toll-like receptor signaling pathway |
| 4668 | TNF signaling pathway |
|  |  |
| **Enriched pathways intersecting Suture IN and Suture OUT (14)** | |
| **GO_id** | **Term** |
| 4014 | Ras signaling pathway |
| 4015 | Rap1 signaling pathway |
| 4060 | Cytokine-cytokine receptor interaction |
| 4062 | Chemokine signaling pathway |
| 4066 | HIF-1 signaling pathway |
| 4151 | PI3K-Akt signaling pathway |
| 4360 | Axon guidance |
| 4510 | Focal adhesion |
| 4512 | ECM-receptor interaction |
| 4514 | Cell adhesion molecules (CAMs) |
| 4630 | Jak-STAT signaling pathway |
| 4670 | Leukocyte transendothelial migration |
| 4810 | Regulation of actin cytoskeleton |
| 5200 | Pathways in cancer |

## Supplementary Table S2. After pathway selection and analysis, the genes involved in all the analysed pathways were first sorted by p<0.05 suture IN vs suture OUT, then the gene lists pooled, duplicate gene IDs removed, and the 25 top most genes sorted by increasing p-value (suture IN vs Suture OUT) panel a, 25 top most genes sorted by FC difference IN-OUT panel b, and the bottom most 25 genes sorted by FC difference IN-OUT panel c, were obtained and are shown in the table below.

| **a:** Sorted by P-value (Suppressed) | | | | | | **b:** Sorted by FC Diff (Suppressed) | | | | | **c:** Sorted by FC Diff (Enhanced) | | | | |  |
| --- | --- | --- | --- | --- | --- | --- | --- | --- | --- | --- | --- | --- | --- | --- | --- | --- |
| **Symbol** | **Fold change 24h** | | | **P-value** | **FC difference** | **Symbol** | **Fold change 24h** | | **P-value** | **FC difference** | **Symbol** | **Fold change 24h** | | **P-value** | **FC difference** | |
|  | **IN** | | **OUT** | **IN vs OUT** | **IN-OUT** |  | **IN** | **OUT** | **IN vs OUT** | **IN-OUT** |  | **IN** | **OUT** | **IN vs OUT** | **IN vs OUT** | |
| *Spry4* | 1.81 | 1.02 | | 1.47E-05 | 0.78 | *Cxcl5* | 52.93 | 11.26 | 3.89E-04 | 41.66 | *Col11a1* | -1.01 | 1.66 | 1.20E-02 | -2.67 | |
| *Bmp4* | 4.50 | 1.54 | | 4.29E-05 | 2.96 | *Ccl2* | 24.75 | 9.15 | 3.64E-03 | 15.60 | *Slit2* | -1.55 | 1.01 | 1.17E-02 | -2.55 | |
| *Lamc2* | 5.49 | 2.17 | | 1.79E-04 | 3.32 | *Timp1* | 30.60 | 18.31 | 1.13E-02 | 12.29 | *Rasa2* | -1.52 | 1.03 | 8.56E-03 | -2.54 | |
| *Serpine1* | 9.05 | 3.64 | | 2.27E-04 | 5.41 | *Il1b* | 16.44 | 6.98 | 3.93E-03 | 9.46 | *Gsk3b* | -1.02 | 1.21 | 1.73E-03 | -2.23 | |
| *Spred3* | 2.10 | 1.28 | | 2.40E-04 | 0.82 | *Cxcr2* | 14.69 | 6.23 | 1.30E-02 | 8.46 | *Lama2* | 1.37 | 2.3 | 4.05E-02 | -0.93 | |
| *Myc* | 2.56 | 1.27 | | 2.46E-04 | 1.29 | *Fgf7* | 9.70 | 2.45 | 3.66E-04 | 7.25 | *Epha7* | 1.41 | 2.26 | 3.14E-03 | -0.85 | |
| *Id1* | 2.05 | 1.17 | | 2.60E-04 | 0.88 | *Sell* | 11.45 | 4.30 | 2.02E-02 | 7.15 | *Sema3c* | -2.33 | -1.49 | 1.63E-02 | -0.84 | |
| *Dusp6* | 3.06 | 1.30 | | 2.90E-04 | 1.76 | *Csf3r* | 12.73 | 5.69 | 3.09E-02 | 7.04 | *Alcam* | 1.23 | 1.89 | 4.07E-04 | -0.65 | |
| *Fgf7* | 9.70 | 2.45 | | 3.66E-04 | 7.25 | *Mmp9* | 12.82 | 6.04 | 1.37E-02 | 6.79 | *Sema3a* | 1.13 | 1.75 | 3.96E-02 | -0.62 | |
| *Cxcl5* | 52.93 | 11.26 | | 3.89E-04 | 41.66 | *Msn* | 16.89 | 10.30 | 3.86E-02 | 6.59 | *Cd36* | 1.11 | 1.67 | 2.93E-02 | -0.56 | |
| *Alcam* | 1.23 | 1.89 | | 4.07E-04 | -0.65 | *Il1r2* | 8.44 | 2.05 | 1.44E-03 | 6.39 | *Plxna4a* | -2.29 | -1.74 | 1.11E-02 | -0.55 | |
| *Cxcl3* | 3.50 | 1.36 | | 4.17E-04 | 2.14 | *Serpine1* | 9.05 | 3.64 | 2.27E-04 | 5.41 | *Phlpp2* | -1.61 | -1.1 | 2.54E-03 | -0.5 | |
| *Wnt7b* | -1.08 | -1.71 | | 4.27E-04 | 0.63 | *Socs3* | 7.13 | 1.83 | 1.34E-03 | 5.30 | *Lpar6* | -1.66 | -1.17 | 7.15E-03 | -0.49 | |
| *Vegfa* | 4.02 | 1.70 | | 4.49E-04 | 2.32 | *Ccl7* | 7.10 | 1.85 | 1.32E-03 | 5.25 | *Col5a2* | 1.1 | 1.59 | 3.25E-02 | -0.49 | |
| *Sema7a* | 2.38 | 1.37 | | 4.78E-04 | 1.01 | *Il24* | 5.49 | 1.17 | 1.30E-02 | 4.32 | *Map2k6* | -1.84 | -1.35 | 1.09E-02 | -0.48 | |
| *Myd88* | 1.84 | 1.09 | | 7.63E-04 | 0.75 | *Cxcl1* | 5.11 | 1.33 | 8.36E-03 | 3.78 | *Robo1* | 1.15 | 1.63 | 7.06E-03 | -0.47 | |
| *Dusp7* | 1.62 | 1.11 | | 9.90E-04 | 0.50 | *Il18rap* | 6.23 | 2.62 | 5.48E-03 | 3.61 | *Arpc5l* | -1.75 | -1.3 | 3.73E-02 | -0.45 | |
| *PVR* | 2.73 | 1.39 | | 1.13E-03 | 1.35 | *Chad* | -3.41 | -6.89 | 2.48E-02 | 3.48 | *Akt3* | 1.48 | 1.92 | 4.09E-02 | -0.44 | |
| *Tgfb1* | 2.25 | 1.37 | | 1.24E-03 | 0.89 | *Lamc2* | 5.49 | 2.17 | 1.79E-04 | 3.32 | *Myh10* | 1.4 | 1.83 | 1.85E-02 | -0.43 | |
| *Ccl7* | 7.10 | 1.85 | | 1.32E-03 | 5.25 | *Fos* | 4.57 | 1.29 | 2.69E-02 | 3.28 | *Fgf13* | 1.1 | 1.52 | 2.02E-02 | -0.42 | |
| *Socs3* | 7.13 | 1.83 | | 1.34E-03 | 5.30 | *Csf2rb* | 5.75 | 2.68 | 6.42E-03 | 3.06 | *Rock2* | -1.62 | -1.24 | 2.18E-02 | -0.38 | |
| *Actn1* | 6.33 | 3.60 | | 1.41E-03 | 2.73 | *Bmp4* | 4.50 | 1.54 | 4.29E-05 | 2.96 | *Vav2* | -1.61 | -1.24 | 6.87E-03 | -0.37 | |
| *Il1r2* | 8.44 | 2.05 | | 1.44E-03 | 6.39 | *Pla2g2a* | -1.64 | -4.55 | 1.74E-02 | 2.91 | *Magi3* | -1.62 | -1.36 | 4.50E-03 | -0.26 | |
| *Efnb1* | -1.36 | -1.78 | | 1.57E-03 | 0.42 | *Dusp1* | 1.72 | -1.13 | 6.70E-03 | 2.85 | *Map4k4* | 1.56 | 1.8 | 3.66E-02 | -0.24 | |
| *Icam1* | 4.88 | 2.87 | | 1.63E-03 | 2.01 | *Nos2* | -1.65 | -4.46 | 2.90E-03 | 2.81 | *Acvr1* | 1.4 | 1.59 | 3.79E-02 | -0.19 | |

**Biological process enrichment analysis.** From biological process enrichment analysis using STRING, only biological processes satisfying multiple testing with p-value < 0.05 were considered for selection and for an-depth downstream analysis. Biological processes analysis by cytoscape BINGO was also performed.

**Supplementary Table S3.** **a,** List of selected biological processes in Suture IN, intersection and in Suture OUT from STRING analysis. **b** is a summary of the biological processes that were identified by cystoscope BINGO in suture IN, intersection and suture OUT. Shown in both A and B is the GO_id number and the biological process term/description.

**a: Biological process** enrichment analysis using STRING

| **Selected biological processes uniquely enriched in Suture OUT (5)** | | |
| --- | --- | --- |
| **GO_id** | **Term** | |
| GO:0006928 | movement of cell or subcellular component | |
| GO:0030155 | regulation of cell adhesion | |
| GO:0002253 | activation of immune response | |
| GO:0050865 | regulation of cell activation | |
| GO:0002694 | regulation of leukocyte activation | |
|  |  | |
| **Selected biological processes uniquely enriched in Suture IN ( 13)** | | |
| **GO_id** | **Term** | |
| GO:0042127 | regulation of cell proliferation | |
| GO:0001944 | vasculature development | |
| GO:0001525 | Angiogenesis | |
| GO:0030335 | positive regulation of cell migration | |
| GO:0001568 | blood vessel development | |
| GO:0048514 | blood vessel morphogenesis | |
| GO:0032103 | positive regulation of response to external stimulus | |
| GO:0060326 | cell chemotaxis | |
| GO:0016337 | single organismal cell-cell adhesion | |
| GO:0002685 | regulation of leukocyte migration | |
| GO:0001775 | cell activation | |
| GO:0050729 | positive regulation of inflammatory response | |
| GO:0042981 | regulation of apoptotic process | |
|  |  | |
| **Selected biological processes intersecting Suture IN and Suture OUT (18)** | | |
| **GO_id** | **Term** | |
| GO:0006955 | immune response | |
| GO:0048583 | regulation of response to stimulus | |
| GO:0071345 | cellular response to cytokine stimulus | |
| GO:0016477 | cell migration | |
| GO:0007155 | cell adhesion | |
| GO:0050776 | regulation of immune response | |
| GO:0048870 | cell motility | |
| GO:0032101 | regulation of response to external stimulus | |
| GO:1903034 | regulation of response to wounding | |
| GO:0006935 | Chemotaxis | |
| GO:0034097 | response to cytokine | |
| GO:0006954 | inflammatory response | |
| GO:0050727 | regulation of inflammatory response | |
| GO:0030334 | regulation of cell migration | |
| GO:0050778 | positive regulation of immune response | |
| GO:0019221 | cytokine-mediated signaling pathway | |
| GO:0002764 | immune response-regulating signaling pathway | |
| GO:0045766 | positive regulation of angiogenesis | |
|  | | |
| **b: Biological processes overrepresentation analysis by Cytoscape** | | |
| **Some of the biological processes uniquely enriched in Suture IN** | | |
| **GO_ID** | | **Description** |
| 19229 | | regulation of vasoconstriction |
| 32680 | | regulation of tumor necrosis factor production |
| 30852 | | regulation of granulocyte differentiation |
| 45601 | | regulation of endothelial cell differentiation |
| 43300 | | regulation of leukocyte degranulation |
| 8064 | | regulation of actin polymerization or depolymerization |
| 30832 | | regulation of actin filament length |
| 10574 | | regulation of vascular endothelial growth factor production |
| 42310 | | vasoconstriction |
| 30833 | | regulation of actin filament polymerization |
|  | | |
| **Some of the biological processes uniquely enriched in Suture OUT** | | |
| **GO_ID** | | **Description** |
| 48858 | | cell projection morphogenesis |
| 904 | | cell morphogenesis involved in differentiation |
| 50679 | | positive regulation of epithelial cell proliferation |
| 2285 | | lymphocyte activation involved in immune response |
| 30307 | | positive regulation of cell growth |
| 48146 | | positive regulation of fibroblast proliferation |
| 30199 | | collagen fibril organization |
| 31344 | | regulation of cell projection organization |
| 10464 | | regulation of mesenchymal cell proliferation |
| 45747 | | positive regulation of Notch signalling |
|  | | |
| **Some of the biological processes intersecting Suture IN and Suture OUT** | | |
| **GO_ID** | | **Description** |
| 16477 | | cell migration |
| 60326 | | cell chemotaxis |
| 1775 | | cell activation |
| 1568 | | blood vessel development |
| 48514 | | blood vessel morphogenesis |
| 45765 | | regulation of angiogenesis |
| 1525 | | angiogenesis |
| 42981 | | regulation of apoptosis |
| 1666 | | response to hypoxia |
| 16337 | | cell-cell adhesion |

## Supplementary Table S4. After biological process selection and analysis, the genes involved in all the analysed biological processes were first sorted by p<0.05 suture IN vs suture OUT, then the gene lists pooled, duplicate gene IDs removed, and the 25 top most genes sorted by increasing p-value (suture IN vs Suture OUT) panel a, 25 top most genes sorted by FC difference IN-OUT panel b, and the bottom most 25 genes sorted by FC difference IN-OUT panel c, were obtained and are shown in the table below.

##

| **a:** Sorted by P-value (Suppressed) | | | | | | **b:** Sorted by FC Diff (Suppressed) | | | | | **c:** Sorted by FC Diff (Enhanced) | | | | |
| --- | --- | --- | --- | --- | --- | --- | --- | --- | --- | --- | --- | --- | --- | --- | --- |
|  | **Fold change 24h** | | | **P-value** | **FC difference** |  | **Fold change 24h** | | **P-value** | **FC difference** |  | **Fold change 24h** | | **P-value** | **FC difference** |
| **Symbol** | **IN** | | **OUT** | **IN vs OUT** | **IN-OUT** | **Symbol** | **IN** | **OUT** | **IN vs OUT** | **IN-OUT** | **Symbol** | **IN** | **OUT** | **IN vs OUT** | **IN-24** |
| *Bmp4* | 4.50 | 1.54 | | 4.29E-05 | 2.96 | *Reg3g* | 34.82 | 9.98 | 7.94E-04 | 24.84 | *Slit2* | -1.55 | 1.01 | 1.17E-02 | -2.55 |
| *Angptl4* | 2.73 | 1.14 | | 1.32E-04 | 1.59 | *Krt16* | 31.71 | 12.43 | 2.63E-03 | 19.28 | *Gsk3b* | -1.02 | 1.21 | 1.73E-03 | -2.23 |
| *Mt2A* | 9.49 | 2.52 | | 1.51E-04 | 6.97 | *Serpinb2* | 23.83 | 8.65 | 1.85E-03 | 15.18 | *Lama2* | 1.37 | 2.3 | 4.05E-02 | -0.93 |
| *Lamc2* | 5.49 | 2.17 | | 1.79E-04 | 3.32 | *Timp1* | 30.60 | 18.31 | 1.13E-02 | 12.29 | *Syne2* | -1.95 | -1.05 | 6.05E-03 | -0.9 |
| *Niacr1* | 4.28 | 1.98 | | 1.82E-04 | 2.30 | *Il1b* | 16.44 | 6.98 | 3.93E-03 | 9.46 | *Epha7* | 1.41 | 2.26 | 3.14E-03 | -0.85 |
| *Otub1* | 1.10 | -1.64 | | 1.82E-04 | 2.75 | *S100a8* | 20.44 | 11.62 | 1.21E-02 | 8.82 | *Col12a1* | 1.2 | 2.05 | 2.42E-02 | -0.85 |
| *Shmt2* | 2.13 | 1.08 | | 2.36E-04 | 1.04 | *Clec4d* | 10.63 | 3.26 | 7.81E-03 | 7.37 | *Sema3c* | -2.33 | -1.49 | 1.63E-02 | -0.84 |
| *Myc* | 2.56 | 1.27 | | 2.46E-04 | 1.29 | *Sell* | 11.45 | 4.30 | 2.02E-02 | 7.15 | *Ptprd* | -1.75 | -1.01 | 6.98E-03 | -0.74 |
| *Id1* | 2.05 | 1.17 | | 2.60E-04 | 0.88 | *Csf3r* | 12.73 | 5.69 | 3.09E-02 | 7.04 | *Notch2* | 1.15 | 1.82 | 3.08E-03 | -0.67 |
| *Dusp6* | 3.06 | 1.30 | | 2.90E-04 | 1.76 | *Mt2A* | 9.49 | 2.52 | 1.51E-04 | 6.97 | *Pla2r1* | -1.97 | -1.31 | 2.67E-02 | -0.66 |
| *Alcam* | 1.23 | 1.89 | | 4.07E-04 | -0.65 | *Rarres2* | 21.45 | 14.59 | 4.14E-02 | 6.86 | *Alcam* | 1.23 | 1.89 | 4.07E-04 | -0.65 |
| *Pglyrp1* | 2.10 | 1.09 | | 4.11E-04 | 1.01 | *Mmp9* | 12.82 | 6.04 | 1.37E-02 | 6.79 | *Sema3a* | 1.13 | 1.75 | 3.96E-02 | -0.62 |
| *Cxcl3* | 3.50 | 1.36 | | 4.17E-04 | 2.14 | *Msn* | 16.89 | 10.30 | 3.86E-02 | 6.59 | *Cdc42bp* | -1.6 | -1.01 | 9.02E-03 | -0.59 |
| *Wnt7b* | -1.08 | -1.71 | | 4.27E-04 | 0.63 | *Il1r2* | 8.44 | 2.05 | 1.44E-03 | 6.39 | *Cd36* | 1.11 | 1.67 | 2.93E-02 | -0.56 |
| *Ass1* | -2.18 | -3.23 | | 4.28E-04 | 1.05 | *Socs3* | 7.13 | 1.83 | 1.34E-03 | 5.30 | *Plxna4a* | -2.29 | -1.74 | 1.11E-02 | -0.55 |
| *Ncoa3* | -1.74 | -1.30 | | 4.62E-04 | -0.44 | *Ccl7* | 7.10 | 1.85 | 1.32E-03 | 5.25 | *Sorbs1* | -1.56 | -1.03 | 2.80E-03 | -0.54 |
| *Fam110c* | 1.01 | -1.67 | | 4.64E-04 | 2.68 | *Il1rl1* | 10.30 | 5.11 | 2.34E-02 | 5.19 | *Ptpn13* | -1.64 | -1.11 | 4.49E-03 | -0.53 |
| *Ccbe1* | 3.08 | 1.81 | | 5.19E-04 | 1.27 | *Egr1* | 5.89 | 1.25 | 5.68E-03 | 4.64 | *Abcb1a* | -1.66 | -1.18 | 1.05E-03 | -0.48 |
| *Adam8* | 1.55 | 1.02 | | 5.40E-04 | 0.52 | *Il24* | 5.49 | 1.17 | 1.30E-02 | 4.32 | *Map2k6* | -1.84 | -1.35 | 1.09E-02 | -0.48 |
| *Mif* | -1.04 | -1.52 | | 6.01E-04 | 0.48 | *Cxcl1* | 5.11 | 1.33 | 8.36E-03 | 3.78 | *Lrrk2* | -1.62 | -1.15 | 4.78E-02 | -0.46 |
| *Arhgap8* | 1.96 | 1.31 | | 6.56E-04 | 0.65 | *Il18rap* | 6.23 | 2.62 | 5.48E-03 | 3.61 | *Ncoa3* | -1.74 | -1.3 | 4.62E-04 | -0.44 |
| *Myd88* | 1.84 | 1.09 | | 7.63E-04 | 0.75 | *Nov* | 4.99 | 1.67 | 1.29E-02 | 3.32 | *Cpd* | -1.58 | -1.16 | 7.87E-03 | -0.43 |
| *Reg3g* | 34.82 | 9.98 | | 7.94E-04 | 24.84 | *Lamc2* | 5.49 | 2.17 | 1.79E-04 | 3.32 | *Myh10* | 1.4 | 1.83 | 1.85E-02 | -0.43 |
| *Hist1h2a* | 1.70 | 1.08 | | 8.10E-04 | 0.62 | *Trem1* | 5.01 | 1.93 | 1.94E-03 | 3.08 | *Cpd* | -1.58 | -1.16 | 7.87E-03 | -0.43 |
| *Spink5* | 1.26 | 1.65 | | 8.84E-04 | -0.40 | *Csf2rb* | 5.75 | 2.68 | 6.42E-03 | 3.06 | *Pcdhb22* | -1.66 | -1.24 | 3.36E-02 | -0.42 |

### Supplementary Table S5. Comparison of gene lists generated from pathway and biological process enrichment analysis.

Pathway vs Biological process

| *Akt3* | *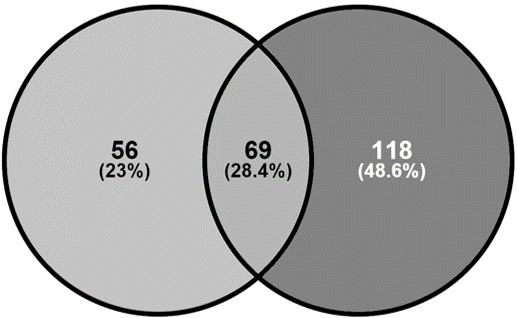* | | | | *Reg3g* | *Ddr1* | *Pla2r1* |
| --- | --- | --- | --- | --- | --- | --- | --- |
| *Fgf7* |  |  |  |  | *Krt16* | *Fam162a* | *Notch2* |
| *Vegfa* |  |  |  |  | *Serpinb2* | *Lynx1* | *Ptprd* |
| *Ngfr* |  |  |  |  | *S100a8* | *Arhgap8* | *Col12a1* |
| *Osmr* |  | *Pla2g2a* | *Wnt7b* |  | *Clec4d* | *Sfrp1* | *Syne2* |
| *Cxcr2* |  | *Fgf13* | *Spi1* |  | *Mt2A* | *Plscr1* | *Cyld* |
| *Ccl2* |  | *Ifngr2* | *Runx1* |  | *Rarres2* | *Hist1h2ak* |  |
| *Il1r1* |  | *Acvr1* | *Ptgs2* |  | *Il1rl1* | *Hpse* |  |
| *Cxcr7* |  | *Il20rb* | *Bmp4* |  | *Egr1* | *Kcnh1* |  |
| *Ralgds* |  | *Il1b* | *Rara* |  | *Nov* | *Plk1* |  |
| *Gngt2* |  | *Tnfrsf12a* | *Map4k4* |  | *Trem1* | *Stk17b* |  |
| *Rasa2* |  | *Il18rap* | *Dusp6* |  | *Otub1* | *Pglyrp3* |  |
| *Magi3* |  | *Tnfrsf1b* | *Dusp1* |  | *Hist1h2ac* | *Adam8* |  |
| *Adora2b* |  | *Csf2rb* | *Irak4* |  | *Fam110c* | *Gipc1* |  |
| *Tnfrsf18* |  | *Ccl7* | *Myd88* |  | *Aurkb* | *Ppif* |  |
| *Ccl20* |  | *Csf3r* | *Ripk3* |  | *Errfi1* | *Snai1* |  |
| *Cxcr5* |  | *Il1r2* | *Bcl3* |  | *Rrm2* | *Ptgfr* |  |
| *Tnfsf13* |  | *Pik3cd* | *Il24* |  | *Niacr1* | *Senp17* |  |
| *Tgfb1* |  | *Shc1* | *Sema3c* |  | *Tubb2b* | *Mif* |  |
| *Cxcl5* |  | *Efna1* | *Slit2* |  | *Gpm6a* | *Degs1* |  |
| *Serpine1* |  | *Id1* | *Mmp9* |  | *Pdpn* | *Spry1* |  |
| *Pfkfb3* |  | *Map2k6* |  |  | *Il36g* | *Zbtb7b* |  |
| *Nos2* |  | *Ccl11* |  |  | *Stfa2* | *Inpp5b* |  |
| *Chad* |  | *Ifnk* |  |  | *LOC498276* | *Wisp2* |  |
| *Mlst8* |  | *Csf3* |  |  | *Nppb* | *Vipr2* |  |
| *Col5a2* |  | *Tnfrsf11a* |  |  | *Ifitm3* | *Il1rn* |  |
| *Col11a1* |  | *Tnfrsf1a* |  |  | *Ptk6* | *Casp4* |  |
| *Stat3* |  | *Il4ra* |  |  | *Angptl4* | *Fntb* |  |
| *Vav2* |  | *Was* |  |  | *Alox5ap* | *Sdf2l1* |  |
| *Rock2* |  | *Jak2* |  |  | *Ptgs1* | *Egr2* |  |
| *Edn1* |  | *Gsk3b* |  |  | *Ccbe1* | *Tes* |  |
| *Phlpp2* |  | *Nos3* |  |  | *Lyve1* | *Klf2* |  |
| *Lamb3* |  | *Timp1* |  |  | *Id3* | *Crtc3* |  |
| *Lpar6* |  | *Cdkn1a* |  |  | *Oas1k* | *Tax1bp3* |  |
| *Actn1* |  | *Ccnd1* |  |  | *Sh2d3c* | *Arhgef3* |  |
| *Cldn4* |  | *Lama2* |  |  | *Ass1* | *Rspo1* |  |
| *Icam1* |  | *Lamc2* |  |  | *Shmt2* | *Cttn* |  |
| *Sele* |  | *Cxcl3* |  |  | *Tinagl1* | *Spon2* |  |
| *Cldn1* |  | *Cxcl1* |  |  | *Myadm* | *Sh2b2* |  |
| *PVR* |  | *Creb3l1* |  |  | *Pglyrp1* | *Fam123b* |  |
| *Selp* |  | *Myc* |  |  | *Apobec3b* | *Inpp5f* |  |
| *Pim1* |  | *Parvb* |  |  | *Plaur* | *Nucb2* |  |
| *Pip4k2c* |  | *Zyx* |  |  | *Runx3* | *Kif13a* |  |
| *Slit3* |  | *Cav2* |  |  | *Rassf6* | *Abcc4* |  |
| *Robo1* |  | *Cd36* |  |  | *Crip2* | *Gpnmb* |  |
| *Efnb1* |  | *Alcam* |  |  | *Nqo1* | *Spink5* |  |
| *Sema6b* |  | *Cd2* |  |  | *Ramp3* | *Tgfbr3* |  |
| *Spred3* |  | *Sdc3* |  |  | *Prg4* | *Nfib* |  |
| *Spry4* |  | *Sell* |  |  | *Epgn* | *Pcdhb22* |  |
| *Arpc5l* |  | *Socs3* |  |  | *Egfl7* | *Cpd* |  |
| *Sema7a* |  | *Msn* |  |  | *Ddah1* | *Ncoa3* |  |
| *Ncf4* |  | *Myh10* |  |  | *Pfn2* | *Lrrk2* |  |
| *Fos* |  | *Sema3a* |  |  | *Zc3h12a* | *Abcb1a* |  |
| *Gadd45g* |  | *Plxna4a* |  |  | *Hmga2* | *Ptpn13* |  |
| *Dusp7* |  | *Epha7* |  |  | *P2ry6* | *Sorbs1* |  |
| *Mlkl* |  | *Unc5b* |  |  | *Mmp28* | *Cdc42bpa* |  |

**Supplementary Figures**


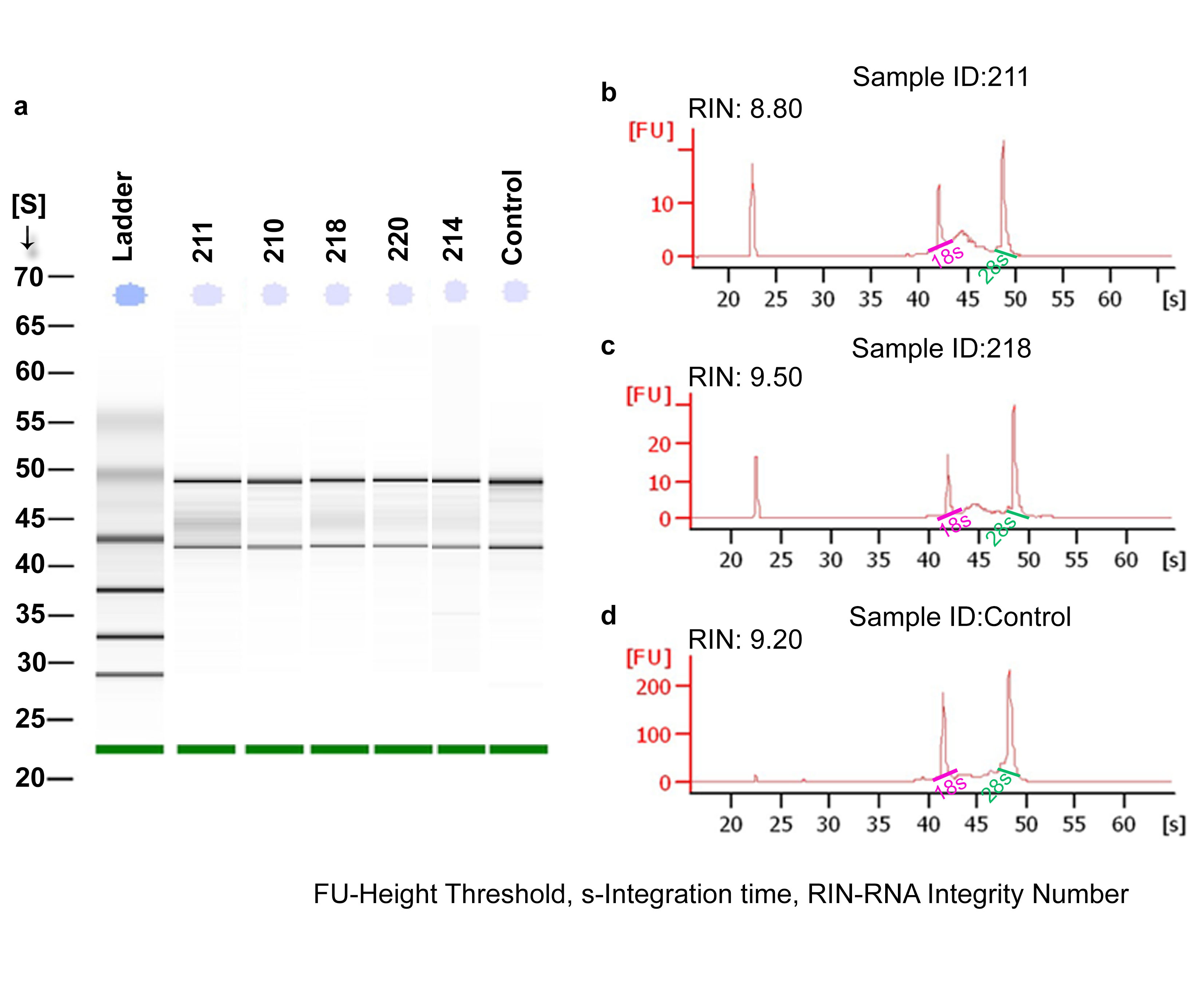


**Supplementary Figure S1.** (**a**) Electrophoretic sizing of selected samples and ladder (RNA 6000 Ladder). (**b-d**) Electropherogram of total RNA for samples; 211, 218 and control respectively. RNA Integrity Number (RIN) of ≥7 was the cut-off for sample inclusion for microarray analysis.


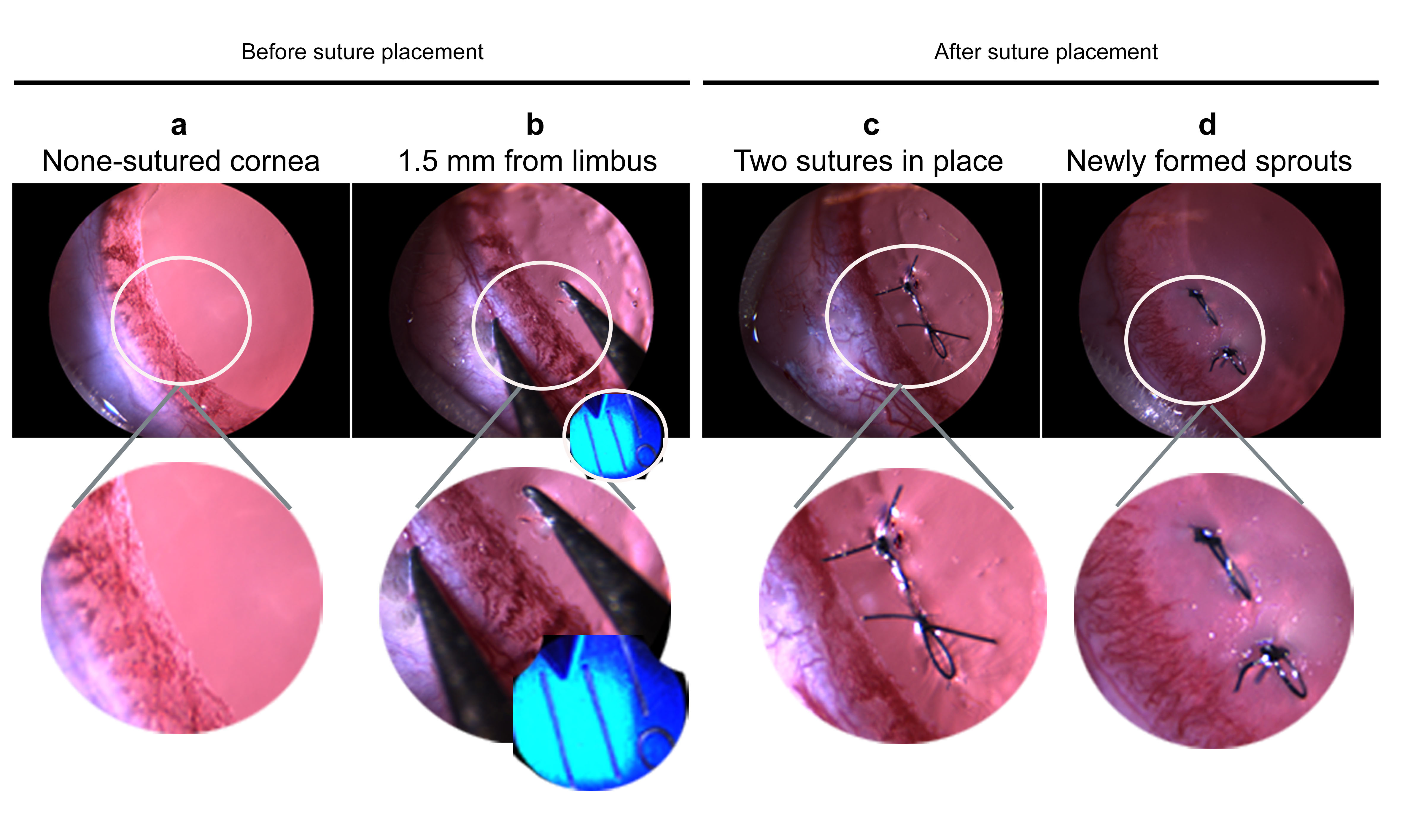
**Supplementary Figure S2. (a)** Non-sutured naive rat cornea, which also serves as a control. **(b)** Vernier calliper measurement (1.5mm) from the limbus towards the centre of the cornea to locate site for suture placement. **(c)** Two sutures placed side by side at the designated location. **(d)** Sprouting towards the suture after four-five days following suture placement.

**
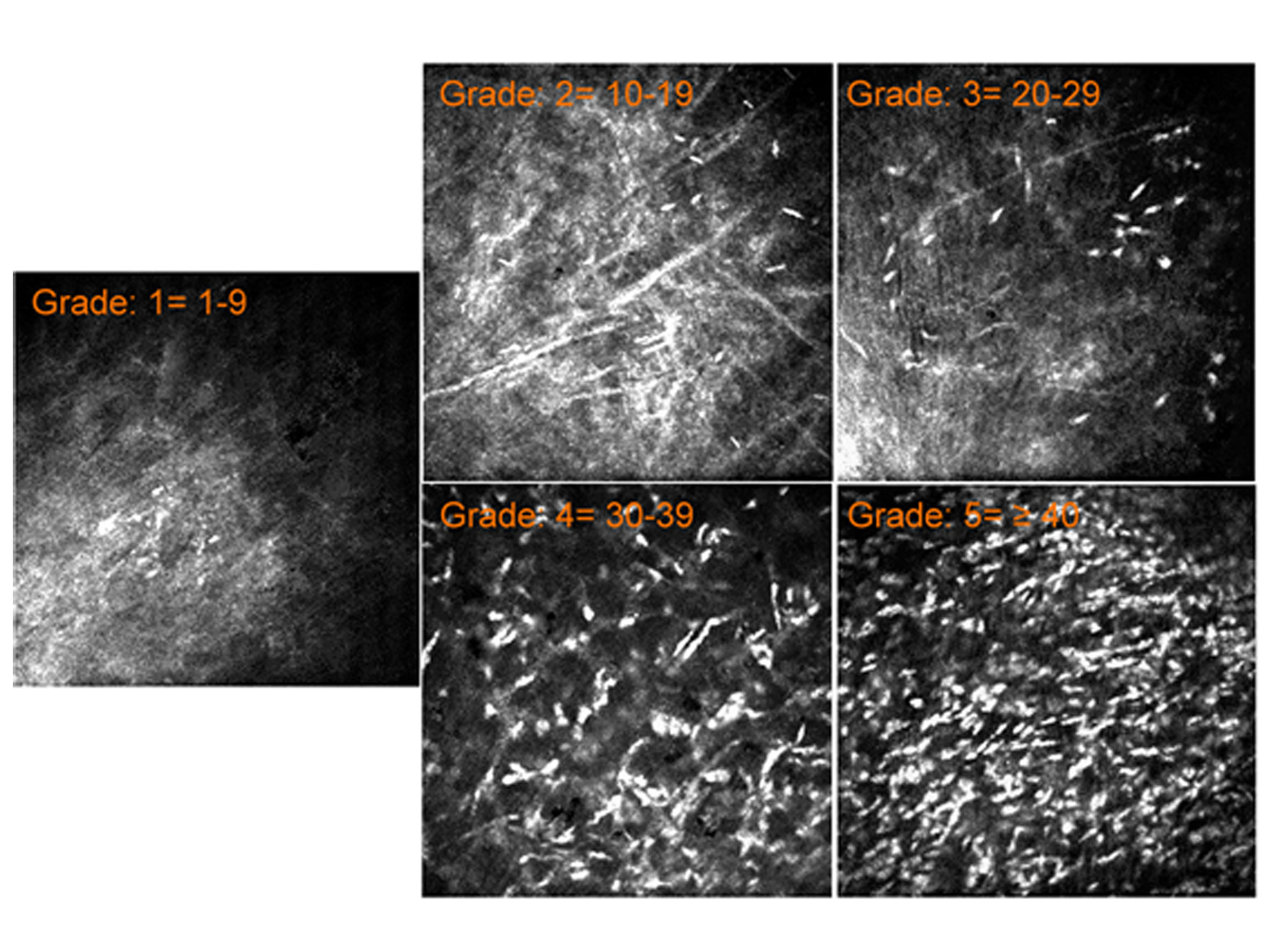
Supplementary Figure S3.** All in vivo confocal microscopy images with granulocytes were collected and the above representative images were extracted to generate the grading scale (1-5).

##
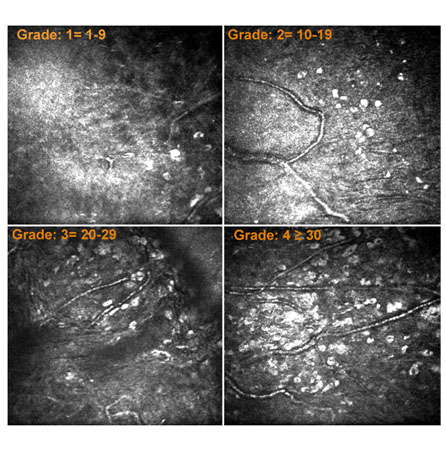
Supplementary Figure S4. All in vivo confocal microscopy images of macrophages were collected and the above representative images were extracted to generate the grading scale (1-4).

**
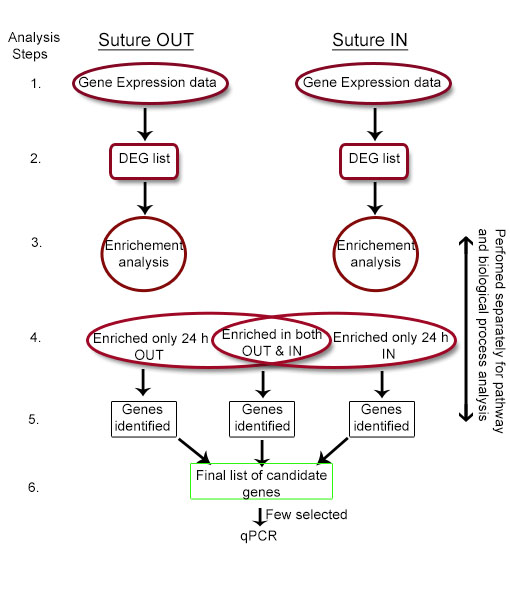
**

### Supplementary Figure S5. Step 1: Microarray raw files are normalised to obtain gene expression data Step 2: From the gene expression data, DEG are obtained by filtering on FC and p-value, both relative to the control. Step 3: The DEGs are enriched into pathways and biological processes. Step 4: The enriched pathways and biological processes are compared between suture IN and suture OUT. Step 5: After selecting pathways and processes of interest, the corresponding genes are identified. Step 6: The identified genes from both pathways and biological processes are pooled, duplicate gene IDs removed, and genes are sorted on p-value suture IN vs Suture OUT, and by FC difference i.e. Suture IN minus suture OUT. The genes are further sorted by q<0.05 relative to the control, to select the number of genes for qPCR validation of fold change.
